# The changing cost‐effectiveness of primary HIV prevention: simple calculations of direct effects

**DOI:** 10.1002/jia2.26494

**Published:** 2025-05-15

**Authors:** Geoff P. Garnett, Joshua T. Herbeck, Adam Akullian

**Affiliations:** ^1^ TB & HIV Team, Bill & Melinda Gates Foundation Seattle Washington USA; ^2^ Institute for Disease Modeling Bill & Melinda Gates Foundation Seattle Washington USA

**Keywords:** cost‐effectiveness analysis, HIV prevention, HIV, incidence, mathematical modelling, number needed to treat

## Abstract

**Introduction:**

Over the course of the HIV pandemic, prevention and treatment interventions have reduced HIV incidence, but there is still scope for new prevention tools to further control HIV. Studies of the cost‐effectiveness of HIV prevention tools are often done using detailed, “transmission‐aware” models, but there is a role for simpler analyses.

**Discussion:**

We present equations to calculate the cost‐effectiveness, budget impact and epidemiological impact of HIV prevention interventions including equations allowing for multiple interventions and heterogeneity in risk across populations. As HIV incidence declines, the number needed to cover to prevent one HIV acquisition increases. Along with the benefits of averting HIV acquisitions, the cost‐effectiveness of HIV prevention interventions is driven by incidence, along with efficacy, duration and costs of the intervention. The budget impact is driven by cost, size of the population and coverage achieved, and impact is determined by the effective coverage of interventions. HIV incidence has declined in sub‐Saharan Africa, making primary HIV prevention less cost‐effective and decreasing the price at which new prevention products provide value. Heterogeneity in risk could in theory allow for focusing HIV prevention, but current screening tools do not appear to sufficiently differentiate risk in populations where they have been applied. The simple calculations shown here provide rough initial estimates that can be compared with more sophisticated transmission dynamic and health economic models.

**Conclusions:**

Simple equations show how the observed declines in HIV incidence in sub‐Saharan Africa make primary prevention tools less cost‐effective. If we require prevention to be more cost‐effective, either we need primary prevention tools to be used disproportionately by those most at risk of acquiring HIV, or they need to be less expensive.

## 1 INTRODUCTION

The adoption of safe sex practices, the scale‐up of antiretroviral treatment and the introduction of biomedical primary prevention interventions such as voluntary medical male circumcision (VMMC) have reduced HIV incidence across sub‐Saharan Africa. Further declines may be possible with the introduction of new HIV prevention products. There is a need to consider what value such new products offer in controlling HIV.

In evaluating the potential benefits of new primary HIV prevention interventions, attention has been given to detailed assumptions about the product profile, pattern of use and epidemiological context, in mathematical models describing the transmission dynamics of HIV [1]. The inclusion of predicted changes in the spread of HIV and the patterns of exposure experienced by cohorts add realism and rigour to such analyses, but also add complexity, as does the detailed consideration of the costs of prevention products and their delivery. The inclusion of such details risks conveying a greater sense of accuracy than is warranted, given the uncertainties surrounding both the behaviours of the products, their patterns of use and the influences on HIV epidemiology.

Detailed mathematical modelling has been extensively used to explore the cost‐effectiveness of primary HIV prevention interventions. Early mathematical models of HIV vaccines, predating the introduction of effective antiretroviral treatment, found that partially efficacy vaccines could be impactful [2, 3]. The subsequent introduction of other interventions has reduced the incidence of HIV and AIDS deaths, reducing the potential impact of such partially effective vaccines. Models have included both generic vaccine properties [4, 5] and properties for specific vaccines including the gp120 vaccine [3], the RV144 vaccine [6, 7] and the HVTN 702 vaccine while they were in phase 3 trials [8, 9]. There have also been models exploring the impact and cost‐effectiveness of vaginal microbicide gels [10], VMMC [11, 12], oral pre‐exposure prophylaxis [13−15], preventive vaginal rings [16, 17] and injectable pre‐exposure prophylaxis [18, 19]. While the detail represented in these analyses influences specific results and recommendations, the resulting complex parameter space may obscure important insights into the fundamental drivers of the impact and cost‐effectiveness of HIV prevention interventions.

It is possible with simple calculations based on patterns of HIV incidence to explore the health economics of new prevention interventions. In the following, simple equations for the cost‐effectiveness, impact and affordability of HIV prevention interventions are defined and their behaviour is illustrated. These analyses allow us to explore the value of introducing new HIV prevention interventions, the price at which prevention products are cost‐effective and the populations for which they would be cost‐effective. The calculations can assist in deciding whether and for whom to introduce a new prevention intervention. Current patterns of incidence for HIV are explored and applied to illustrate the use of the models.

## 2 DISCUSSION

### 2.1 Cost‐effectiveness, impact and affordability calculations

The immediate impact and cost‐effectiveness of any primary HIV prevention intervention is a function of the expected HIV incidence in the intervention's absence. The higher the risk of HIV acquisition, the fewer the number of people using the prevention method needed to prevent an acquisition. The number needed to treat (NNT) is normally calculated comparing the control and treatment arms of trials [20], where in the case of HIV prevention “treatment” would refer to the prevention intervention. Here, we will use the number needed to *cover* (NNC) as a more intuitive nomenclature for prevention. The NNC can be derived if we know the efficacy of the intervention (*e*), the per susceptible incidence per unit of time (e.g. per year) without the intervention (*I*) and the duration of protection in the same units (e.g. years) (*d*):

(1) NNC=1/e.I.d.

This number needed to cover (NNC) is central to calculating the cost‐effectiveness of an intervention. The cost per acquisition averted is:

(2) C=NNC.K,

where *K* is the average cost of the prevention intervention. The average cost‐effectiveness can be expressed as the cost per disability adjusted life‐years (DALY) averted (*C_d_*):

(3) $$C_{d}=\left(NNC.K-T\right)/D.$$

Here, *T* is the lifetime treatment costs for a person living with HIV (PLHIV), and *D* is the DALYs associated with an HIV acquisition. The treatment costs and DALYs associated with an HIV acquisition will change according to age and over time but for simplicity, we use approximate average values.

The effective coverage of prevention (i.e. the proportion correctly using the intervention) does not influence cost‐effectiveness but does influence the affordability and the impact. Affordability over a year for the provider is determined by the budget impact (*B*) per year of an intervention. This is the costs of scaling up the intervention to a given effective coverage over a year (*f*):

(4) $$B=k.f.Pop.\left(1-P\right).$$

In this case, *k* is the costs per person per year of the prevention intervention. *Pop* is the size of the population and *P* is the prevalence of infection (as a proportion). Additionally, over the long term, the budget would be affected by reduced treatment costs.

Impact (*A*), the number of new acquisitions averted per year, is defined by:

(5) $$A=I.f.e.Pop.\left(1-P\right).$$

When combining more than one approach to prevention, the residual incidence following the effects of one intervention should be applied in calculating the number needed to treat to prevent an acquisition using the second intervention:

(6) $$NNC2=1/e_{2}.d_{2}\left(1-f_{1}.e_{1}\right).I,$$

where *e_1_* and *f_1_* are the efficacy and coverage of first intervention over the duration of the second intervention *d_2_*, and *e_2_* is the efficacy of the second intervention. This allows us to calculate a value analogous to the incremental cost‐effectiveness ratio for the second intervention (C2). NNC1 is the number needed to cover for the first intervention and *k*2 and *k*1 are the average cost per person of the two interventions:

(7) $$C2=\frac{NNC2.k2}{NNC1.k1}.$$

With multiple interventions, each should be considered in turn with the more cost‐effective interventions considered first. This assumes that one intervention does not replace another.

In theory, heterogeneity in risk allows a different impact and cost‐effectiveness in sub‐populations with different risk of HIV acquisition, such that the number needed to cover in risk group *i* (*NNC_i_*) is:

(8) $$NNC_{i}=1/e.d.I_{i}.$$

If coverage includes multiple risk groups of susceptibles, then the relative size of each risk group, *g_i_*, and the fraction of those covered in each, *f_i_*, matters for the aggregate number needed to cover (NNC), which is a function of the proportion covered falling into each risk group:

(9) $$NNC=1/1e.d.\sum_{i=1}^{n}\left(\frac{g_{i}.f_{i}}{\sum_{j=1}^{n}g_{j}.f_{j}}\;.I_{i}\right)e.d.\sum_{i=1}^{n}\left(\frac{g_{i}.f_{i}}{\sum_{j=1}^{n}g_{j}.f_{j}}\;.I_{i}\right).$$

The coverage of the risk group, *f_i_*, and actual size of the risk group, *Pop_i_*, matters for impact over a year:

(10) $$A=e.\sum_{i=1}^{n}\left(f_{i}.I_{i}Pop_{i}\left(1-P_{i}\right)\right).$$

**Figure 1:**
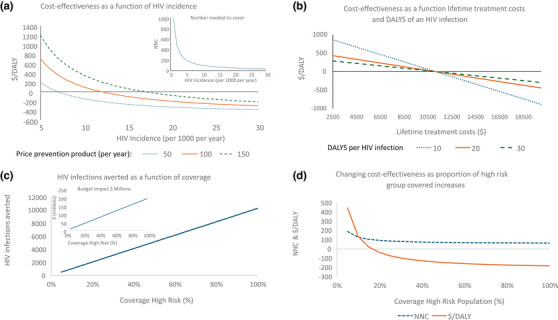
Cost‐effectiveness calculation for primary HIV prevention. (a) The $/DALY of a 90% efficacious prevention product for three prices (per person per year), as a function of HIV incidence (Equation 2). An additional $20 per year cost of delivery is included, lifetime HIV treatment costs are assumed to be $10,000 and someone living with HIV incurs 20 DALYs. The insert shows the relationship between NNC for a year to prevent one HIV acquisition and incidence per year for a product with 100% efficacy (Equation 1). (b) The relationship between the cost‐effectiveness ($/DALY) and lifetime HIV treatment costs and DALYs (Equation 2). Incidence is 1%, efficacy 90%, price per year $80 and costs of delivery $20 per year. (c) The impact of coverage on people newly acquiring HIV (Equation 8) for a population of 10 million where 90% are in a “low‐risk” group with a prevalence of 20% and incidence of 0.1% and 10% in a “high‐risk” group with prevalence of 50% and an incidence of 2%. This represents a population in which incidence has been declining. Without prevention, there are 17,200 infections per year. Coverage of the high‐risk group is illustrated, with one‐fifth that coverage assumed for the low‐risk group. The insert shows the budget impact in millions of dollars as a function coverage for this population for prevention with a price of $80 and delivery costs of $20 per year. (d) For the same heterogenous population, average cost‐effectiveness is shown as a function of coverage of the high‐risk population. Coverage of the low‐risk population is kept at 1%. Efficacy 90%, lifetime HIV treatment costs $10,000, DALYs associated with HIV 20, price of product and delivery costs $80 and $20 per year.

The values derived from these simple equations are explored in Figure 1.

As incidence declines, the number needed to cover and the cost of preventing someone from acquiring HIV increases dramatically. These costs can be offset by lifetime HIV treatment costs if high or the number of DALYs associated with a person living with HIV.

Both the impact on HIV and the budget impact are linear functions of prevention coverage. It costs more to prevent more acquisition of HIV. However, the more focused HIV prevention is on those with a high risk of acquiring acquisition, the better cost‐effectiveness will be.

A review of estimates of incidence was undertaken at the end of 2023 (between 22nd December 2023 and 2nd January 2024) and from 2010 to 2019 identified 291 studies covering 22 African countries [21]. HIV incidence can either be measured directly in cohort studies or by using tests for recent acquisition in cross‐sectional surveys. Most published estimates of directly measured incidence are a few years out of date. The analysis found significant declines in HIV incidence over the period. For the general population, incidence estimates ranged from 0.35 to 3.28 per hundred person years. Population‐based cohorts from Karonga in Malawi, Kisesa in Tanzania, Manicaland in Zimbabwe, Masaka in Uganda, Rakai in Uganda and uMkhanyakude in South Africa were analysed and show incidence among different age groups of men and women declining over time between 2000 and 2017 from around 3 to less than 1%. The highest incidence was observed in uMkhanyakude, which fell steeply, but late on in the period of the analysis [22]. In evaluating the DREAMS project, HIV incidence among 15‐ to 24‐year‐old women in uMkhanyakude was observed over the period 2016−2018 and found to be 2.8 per hundred person years among 15‐ to 19‐year‐olds and 5.8 among 20‐ to 24‐year‐olds. This compares to the rates observed for Gem in Kenya, where the incidence among 20‐ to 24‐year‐old women was 0.64 per hundred person years [23].

Exploring the implications of these incidence patterns, we can calculate the number needed to cover with HIV prevention to avert one HIV acquisition. Assuming 100% efficacy, this number would be 17 among 20‐ to 24‐year‐old women in uMkhanyakude, whereas for the same age group in Gem, it would be 167. The estimates based on the systematic review for the general population would be as high as 285 and as low as 30.

### 2.2 National level cost‐effectiveness

UNAIDS estimates people newly acquiring HIV per year through fitting models to HIV prevalence data [24]. Estimates of incidence across all ages for each country have been published [25]. Assuming this incidence occurs among adults, we can calculate the incidence for those adults by dividing the all‐age incidence by the fraction of the population that is adult (aged 16–64 years from the World Bank estimates). To allow for those already living with HIV, we subtract the prevalence of HIV from adults so that the incidence is applied to those susceptible [25]. Using Equations (1) and (2), we can use these estimates to calculate the costs per acquisition averted. Here, for illustration, we assume the prevention intervention costs $50 per person and has a 90% efficacy. The resulting costs per acquisition averted in Africa range from $3300 for Eswatini with an incidence per 1000 of 7.65 and a prevalence of 27.8% to $525,150 for Eritrea with an incidence of 0.06 per 1000 and prevalence of 0.5%. In South Africa, the cost would be $7150 per acquisition averted, in Botswana $8300, Zambia $12,550, Zimbabwe $18,200, Malawi $24,900 and Kenya $43,100. These costs per acquisition averted use 2022 estimates, and if incidence falls further, they will increase.

### 2.3 Heterogeneity in risk

The UNAIDS estimates are for the whole population where in most countries incidence is below 1% and often below 0.5%. For some specific age groups of women in high‐risk locations such as KwaZulu Natal, South Africa, or among young sex workers and men who have sex with men (MSM), it can be much higher. If we can identify those who are most at risk in a population, then we can focus our HIV prevention. Some studies have tried to identify risk factors for HIV acquisition and have been able to divide populations according to risk scores depending on these variables [26, 27]. Such analyses allow us to estimate the fraction of the population among which most acquisitions fall. In the study of Kagaayi and colleagues [26] from Uganda in 2003−2011 where incidence rates were 0.98 (0.86−1.12) for men, and 1.11 (1.0−1.24) for women, among men 55.1%, 23.6% and 22.3% of acquisitions and for women 48.0%, 20.7% and 31.3% of acquisitions were in the top quarter, the second quarter and bottom half of the population according to risk scores. This would yield an average NNC for men of 102 and for women of 90 but 46 and 47 for the quarter of the population within which half of acquisitions occur.

Trials such as the VOICE trial (2009−2011) have recruited women with high HIV incidence. Women in the VOICE trial were heterogeneous and were classified with a risk score [27]. Women with a risk score of 6 and above comprised 52% of the women and accounted for 80% of HIV acquisitions. These women had an incidence of 9.6% and yield a number needed to cover of 10.4.

Heterogeneity in HIV incidence should allow for focusing HIV prevention interventions on certain population groups, but there are questions about how feasible this is. Is it possible to identify generalizable risk factors that provide enough discriminatory power to allow cost‐effective targeting, and how can this targeting be done without stigmatizing or screening out those who can benefit?

### 2.4 Limitations of simple calculations

Our analysis makes simplifying assumptions in calculating epidemiologic impacts and costs. One of the most important is the lack of indirect effects of the interventions. Preventing the acquisition of HIV averts not only the infection and disease in the person directly but also averts other subsequent acquisitions that would derive from the PLHIV. Calculations of benefits only among those directly protected will underestimate the effects of prevention and the scale of this underestimation will differ between populations. It is most important to consider the influence of transmission dynamics on cost‐effectiveness when the qualitative conclusions from the calculation could be altered. Because HIV epidemics play out over decades rather than weeks and months, the non‐linear dynamics of HIV have less influence on health economic analyses than those for many short‐lived infectious diseases. If the cost‐effectiveness of HIV prevention in our analysis is well above an accepted threshold, then it is unlikely that including onward transmission will change conclusions. More work is needed to systematically compare linear models of HIV control with transmission dynamics models to better understand when the former are adequate [28].

For simplicities sake, we use averages for the costs of HIV prevention interventions, HIV treatment costs and DALYs associated with living with HIV—all of these will vary across populations. While incidence is implicitly part of any cost‐effectiveness analysis, it is explicit in our calculations and is subject to uncertainty and change over time.

There is a question about the precision and accuracy needed to help policymakers make better decisions. When is confidence in results sufficient to allow decisions and what is the value of providing more precise results? The more expensive HIV prevention tools are, the more accurately they will need to be focussed to be cost‐effective. However, there is a cost to generating the information needed to focus on HIV prevention interventions, and we should consider when this cost outweighs the improved efficiency that is possible.

## 3 CONCLUSIONS

The main drivers of the cost‐effectiveness of HIV prevention interventions are HIV incidence, the efficacy and duration of protection, and the cost of the use of the intervention. Simple calculations allow a rapid assessment of the relationship between the characteristics of an HIV prevention intervention and its cost‐effectiveness, impact, and affordability [29]. Use in those with a greater risk makes interventions more cost‐effective, but our current approaches for targeting in sub‐Saharan African populations do not allow for substantial gains in efficiency.

## COMPETING INTERESTS

GPG, JTH and AA are all employees of the Bill and Melinda Gates Foundation.

## AUTHORS’ CONTRIBUTIONS

GPG conceived the commentary, developed the equations, performed the calculations and drafted the manuscript. JTH and AA provided input on the equations and calculations and revised the manuscript.

## DISCLAIMER

The views expressed in this article are those of the authors and do not necessarily represent those of the Bill and Melinda Gates Foundation.

## Data Availability

All the data analysed in this paper was taken from the published literature.
